# Terrain Adaptability Mechanism of Large Ruminants' Feet on the Kinematics View

**DOI:** 10.1155/2015/151686

**Published:** 2015-12-16

**Authors:** Qun Zhang, Xilun Ding, Kun Xu

**Affiliations:** ^1^Space Robot Laboratory, School of Mechanical Engineering and Automation, Beihang University, Beijing 100191, China; ^2^State Key Laboratory of Robotics and System, HIT, Harbin 150001, China

## Abstract

Ruminants live in various parts of land. Similar cloven hooves assist ruminants in adapting to different ground environment during locomotion. This paper analyzes the general terrain adaptability of the feet of ruminants using kinematics of the equivalent mechanism model based on screw theory. Cloven hooves could adjust attitude by changing relative positions between two digits in swing phase. This function helps to choose better landing orientation. “Grasping” or “holding” a rock or other object on the ground passively provides extra adhesion force in stance phase. Ruminants could adjust the position of the metacarpophalangeal joint or metatarsophalangeal joint (MTP or MCP) with no relative motion between the tip of feet and the ground, which ensures the adhesion and dexterity in stance phase. These functions are derived from an example from chamois' feet and several assumptions, which are believed to demonstrate the foundation of adaptation of ruminants and ensure a stable and continuous movement.

## 1. Introduction

Animals have evolved the feet of various shapes and functions to adjust to complex terrains. Insects' feet have small spines and hooks that help them to climb [1], while geckos have the ability to climb vertical surfaces like walls and even ceilings, using five hundred thousand keratinous hairs [2, 3]. Mammals have less diversity than insects and reptiles. Ruminants (Artiodactyl) that have larger cloven hoof than most insects and geckos, including cattle, goat, camel, and deer, have a wide distribution on the earth. Despite the various living environments, ruminants have a similar foot structure [4, 5], covered by neither setae nor hook, which is quite different from that of insects and reptiles. With the assistance of simple, reliable, and strong feet, they adjust to the terrain on which they feed, mate, and avoid predators very well. Buffalo and cattle can go through mud easily [6]. Camels have special and soft feet to cross deserts [7]. Goats and blue sheep can climb up and down cliffs and ledges steadily and fleetly to feed on any grass, shrub, or trees [8, 9]. Their cloven hooves can spread apart when contacting the ground and “grasp” the rock to avoid slip [10, 11]. Previous studies [12–15] take the foot as a whole to discuss the moving characteristics and no theoretical analysis of kinematics of the foot is elucidated. Indeed, the foot is very complicated, constituted by skeleton, multiple joints, ligament, muscles, subcutis, and some skin modifications [16]. We believe that the similar structure forms the basis of adaptability of ruminants during locomotion. The theoretical analysis of kinematics of the foot reveals moving ability and dexterity in general and makes us understand more about the terrain adaptability of large ruminants.

This paper aims to figure out the general functions of the feet as follows: how the feet could adjust the attitude in swing phase and how the feet could keep the dexterity and adhesion in stance phase. An equivalent mechanism model of the foot is built based on the skeleton and joints of the foot. Using the mechanism kinematics and screw theory, we could determine the workspace of the mechanism as a dexterity indicator. Finally, we employ the length of digits' bones of chamois on the theoretical model to discuss the terrain adaptability of the ruminants.

## 2. Methods

### 2.1. Structure of Ruminants' Feet

**Figure 1:**
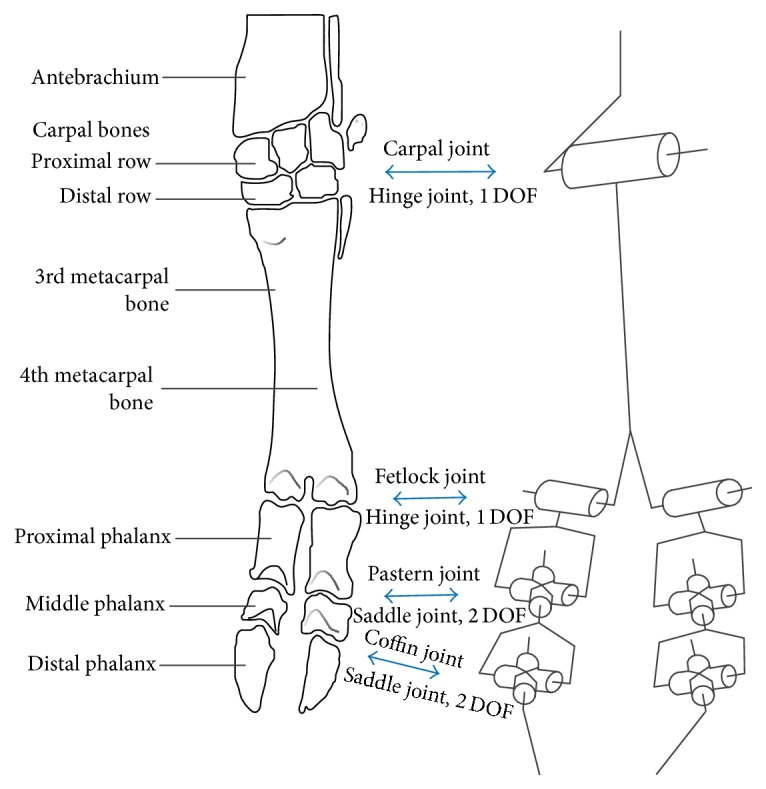
The skeleton and articulation of the ox's manus (schematic), which can be equivalent to an articulated mechanism.

The skeletons and joints of ruminants' manus and tarsus are similar, but they vary in size, such as bovine [17], camel [18], and sheep [19]. The structure of ruminants' manus is shown in Figure 1 [16]. The skeleton of the manus consists of carpal bones, metacarpal bones, and phalanges. In ruminants, there are two digits left. Each digit has three phalanges. The carpal joint (MCP in forelimb) is composite articulations. The carpal joint acts as a hinge joint. Due to the complex anatomy of the carpal skeleton complemented by numerous ligaments of the carpus, the primary movements of the carpal joint are flexion and extension [16]. In ruminants, the remaining third and fourth metacarpal bones are fused and no movement is possible [4]. The two fetlock joints are hinge joints that can only flex and extend. The pastern joints are saddle joints due to the concavo-convex shape of the joint surfaces. They act mainly as hinge joints [20]. However, served as saddle joints, pastern joints are biaxial joint, allowing flexion and extension and a limited range of lateral movements [16]. The coffin joints are similar to the pastern joints. Tarsal joint (MTP in the hindlimb) is also composite joints. The tarsal bones and joints are different from the corresponding ones in the forelimb [4], while the bones and joints of the metatarsus and digits are similar.

**Figure 2:**
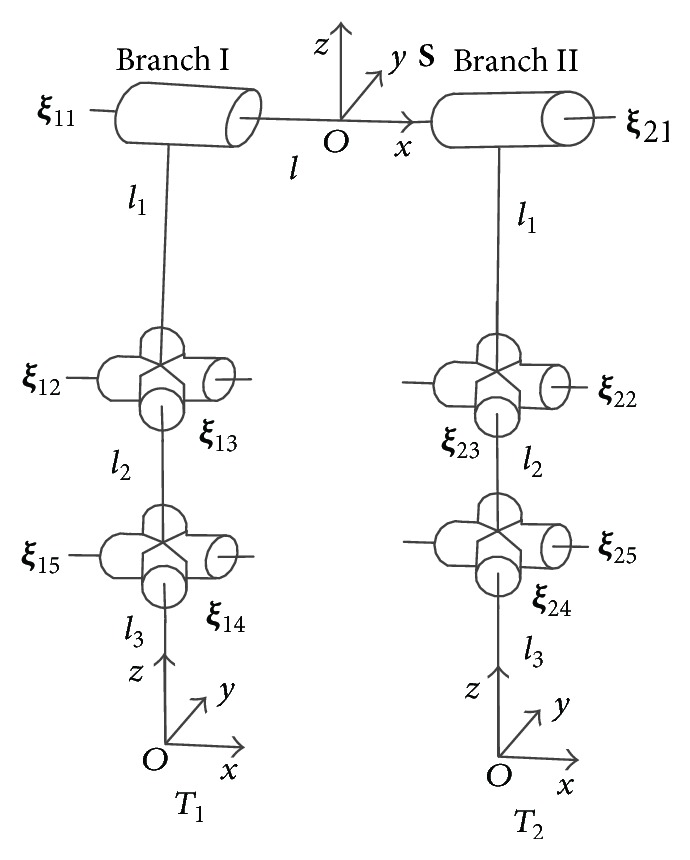
The mechanism of foot in swing phase (reference configuration).

Due to the anatomy in Figure 1 (modified from [21]), we can draw diagram of a mechanism. The revolute joint connects the two same branches; each one consists of one revolute joint and two universal joints. Since the two branches contact the ground in stance phase, we just discuss the mechanism constituted by the two branches. That is, we focus on the functions of two digits, which are essentially the same in the manus and tarsus. In the following part, we define phalanxes and corresponding joints as foot (as shown in Figure 2).

### 2.2. Observations of Goat's Feet

The feet is observed from three adult female goats (*Capra hircus*, breed), ranging in body mass from 23 to 28 kg. All goats were from Beijing Badaling Safari World and apparently healthy. Under the approval of Beijing Badaling Safari World and the professional guidance and assistance of the staffs, the goats were made to lie on their side. The metacarpal bones or metatarsal bones were grabbed by two of the staff to provide a closer observation. Thus, unaffected by the movement of the upper limb, the movement of feet was scrutinized. The goats were examined and released very fast to avoid stress and injury.

### 2.3. Kinematics in Swing Phase

The mechanism in Figure 2 is open chain mechanism, composed of a fixed platform at the top connected two branches, which can move freely in the swing phase. Both branch I and branch II consist of three serially connected joints: one revolute joint and two universal joints. The universal joints are modeled as two revolute joints intersecting at one point. In the joint notation **ξ**_*ij*_ (also screw notation), the first subscript *i* denotes the branch number and the second subscript *j* denotes the joint number within the branch. *θ*_*ij*_ ∈ *R* (*i* = 1,2,…, 5; *j* = 1,2) denotes the amount of the joint **ξ**_*ij*_ rotation, and *L*_*ij*_ ∈ *R* ^3×1^ (*i* = 1,2, 3; *j* = 1,2) denotes the link of the branch.

According to screw theory [22, 23], the twist coordinates of kinematic pair are **ξ**(**v**, **ω**) ∈ *R* ^6×1^, where **ω** ∈ *R* ^3×1^ is the axis of rotation and **v** = −**ω** × **q** (**q** ∈ *R* ^3×1^ is a point on the axis) if the joint is a revolute joint.

The cross product by **ω** is a linear operator, and **ω** × **q** can be represented using a matrix:

(1) $$\begin{array}{c} \omega \times q=\hat{\omega }q=\left[\begin{array}{ccc} 0 & -\omega_{3} & \omega_{2}\\ \omega_{3} & 0 & -\omega_{1}\\ -\omega_{2} & \omega_{1} & 0 \end{array}\right]q. \end{array}$$

The 4 × 4 matrix $\hat{\xi }$ given in (2) is the generalization of the skew-symmetric matrix $\hat{\omega }\in so(3)$

(2) $$\begin{array}{c} \hat{\xi }=\left[\begin{array}{cc} \hat{\omega } & v\\ 0 & 0 \end{array}\right]. \end{array}$$

We let ***θ*** = 0 correspond to the fully extended configuration and attach base and tool frames as shown in Figure 2. Due to the product of exponentials formula for the manipulator forward kinematic, the transformation between tool and base frames in branch I is given by

(3) $$\begin{array}{c} g_{st}^{b1}\left(\;\theta \;\right)=e^{{\hat{\xi }}_{11}\theta_{11}}e^{{\hat{\xi }}_{12}\theta_{12}}e^{{\hat{\xi }}_{13}\theta_{13}}e^{{\hat{\xi }}_{14}\theta_{14}}e^{{\hat{\xi }}_{15}\theta_{15}}g_{st}^{b1}\left(\;0\;\right), \end{array}$$

where *g*_*st*_^*b*1^(0) refers to the transformation between tool and base frames at ***θ*** = 0.

The transformation between tool and base frames in branch II is similar

(4) $$\begin{array}{c} g_{st}^{b2}\left(\;\theta \;\right)=e^{{\hat{\xi }}_{21}\theta_{21}}e^{{\hat{\xi }}_{22}\theta_{22}}e^{{\hat{\xi }}_{23}\theta_{23}}e^{{\hat{\xi }}_{24}\theta_{24}}e^{{\hat{\xi }}_{25}\theta_{25}}g_{st}^{b2}\left(\;0\;\right). \end{array}$$

### 2.4. Kinematics in Stance Phase

**Figure 3:**
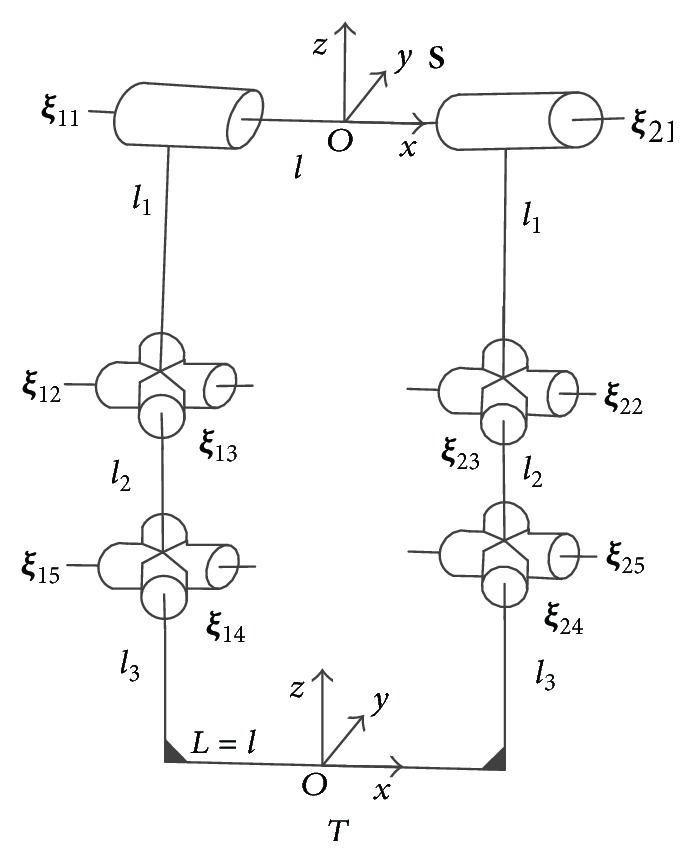
The mechanism of grasping rock passively (reference configuration).

After regulating the attitude of the two digits in swing phase, ruminants can embed the protrusion of rock between their digits in stance phase to increase the adhesion and remain stable. However, odd toed mammals like horse cannot seize a stone by their odd digit. There are interdigital ligaments (cruciate ligament) between the two digits (the space between two claws is called interdigital cleft), which are not found in the digit of horse [4]. Distal interdigital ligament bridges middle phalanx and distal phalanx of two digits. When the rock is embedded in the interdigital cleft, the foot will splay out. Since the interdigital ligaments limit this movement [16], the two digits will tend to close. This is like “grasping” or “holding” the rock except that it is passive. When ruminants firmly “grasp” a rock in stance phase, the relative position between two digits remains unchanged. This situation resembles “grasping” an object using two manipulators. The mechanism transforms into the single loop mechanism with grasping constraint (Figure 3). The mechanism is composed of a moving platform at the bottom connected to a fixed platform at the top by two branches.

#### 2.4.1. DOF in the Reference Configuration

*Branch I*. The branch motion-screw system {S_b1_} = {**ξ**_11_, **ξ**_12_, **ξ**_13_, **ξ**_14_, **ξ**_15_} is described by its basis

(5) $$\begin{array}{c} \left\{\;S_{b1}\;\right\}=\left\{\begin{array}{c} \xi_{11}={\left(0,0,0,1,0,0\right)}^{T}\\ \xi_{12}={\left(0,-l_{1},0,1,0,0\right)}^{T}\\ \xi_{13}={\left(l_{1},0,-\frac{l}{2},0,1,0\right)}^{T}\\ \xi_{14}={\left(l_{1}+l_{2},0,-\frac{l}{2},0,1,0\right)}^{T}\\ \xi_{15}={\left(0,-l_{1}-l_{2},0,1,0,0\right)}^{T} \end{array}\right\}. \end{array}$$

Constraint-screw **F**(**f**, **τ**) and its motion-screw **ξ**(**v**, **ω**) are related by

(6) $$\begin{array}{c} F\cdot \xi =0, \end{array}$$

where $F=\left[\begin{array}{c} f\\ \tau \end{array}\right]$, $\xi =\left[\begin{array}{c} v\\ \omega \end{array}\right]$.

According to (6), a basis for branch **S**_*b*1_^*r*^ constraint-screw system can be calculated

(7) $$\begin{array}{c} \left\{\;S_{b1}^{r}\;\right\}=\left\{\begin{array}{c} F_{11}={\left(0,0,\frac{2}{l},0,1,0\right)}^{T}\\ F_{12}={\left(0,0,0,0,0,1\right)}^{T} \end{array}\right\}. \end{array}$$

*Branch II*. The branch motion-screw system {**S**_*b*2_} = {**ξ**_21_, **ξ**_22_, **ξ**_23_, **ξ**_24_, **ξ**_25_} is described by its basis

(8) $$\begin{array}{c} \left\{\;S_{b2}\;\right\}=\left\{\begin{array}{c} \xi_{21}={\left(0,0,0,1,0,0\right)}^{T}\\ \xi_{22}={\left(0,-l_{1},0,1,0,0\right)}^{T}\\ \xi_{23}={\left(l_{1},0,\frac{l}{2},0,1,0\right)}^{T}\\ \xi_{24}={\left(l_{1}+l_{2},0,\frac{l}{2},0,1,0\right)}^{T}\\ \xi_{25}={\left(0,-l_{1}-l_{2},0,1,0,0\right)}^{T} \end{array}\right\}. \end{array}$$

A basis for branch **S**_*b*2_^*r*^ constraint-screw system can be calculated

(9) $$\begin{array}{c} \left\{\;S_{b2}^{r}\;\right\}=\left\{\begin{array}{c} F_{21}={\left(0,0,-\frac{2}{l},0,1,0\right)}^{T}\\ F_{22}={\left(0,0,0,0,0,1\right)}^{T} \end{array}\right\}. \end{array}$$

Dai et al. proposed a generalized Kutzbach-Grübler mobility criterion [24] to calculate the degrees of freedom (DOFs) *M* for *n* bodies connected by *g* joints, each with *f*_*i*_ degrees of freedom

(10) $$\begin{array}{c} M=d\left(\;n-g-1\;\right)+\overset{g}{\underset{i=1}{\sum }}f_{i}+v-\zeta , \end{array}$$

where *d* is the order of mechanism, *v* represents redundant constraint, and *ζ* is passive DOF. For single loop mechanism, *v* is equal to 0 [25]

(11) $$\begin{array}{c} d=6-\lambda , \end{array}$$

where *λ* is common constraint.

Thus, at the reference configuration in Figure 3,

(12) $$\begin{array}{c} \lambda =\dim\left(\;\left\{\;S_{b1}^{r}\;\right\}\cap \left\{\;S_{b2}^{r}\;\right\}\;\right)=1. \end{array}$$

The degrees of freedom of the mechanism are drawn

(13) $$\begin{array}{c} M=5\times \left(\;6-6-1\;\right)+\left(\;1+1+2+2+2+2\;\right)=5. \end{array}$$

When the mechanism is not at the reference configuration, generally,

(14) $$\begin{array}{c} \lambda =\dim\left(\;\left\{\;S_{b1}^{r}\;\right\}\cap \left\{\;S_{b2}^{r}\;\right\}\;\right)=0. \end{array}$$

Thus, the degrees of freedom of the mechanism are drawn

(15) $$\begin{array}{c} M=6\times \left(\;6-6-1\;\right)+\left(\;1+1+2+2+2+2\;\right)=4. \end{array}$$

#### 2.4.2. Inverse Kinematics

The *XYZ* Euler angles (*α*, *β*, *γ*) are available for describing the orientation of the moving platform relative to the base and $\left[\begin{array}{ccc} x & y & z \end{array}\right]$ refer to origin coordinates of moving platform. Through constraint-screw systems  {**S**_*b*1_^*r*^} and {**S**_*b*2_^*r*^}, moving platform cannot rotate about *z*-axis in the reference configuration. So, the angles (*α*, *β*) can describe the orientation of the moving platform of the mechanism. We define that *α* represents the roll angle, *β* refers to the pitch angle, and *γ* refers to the yaw angle. $\left[\begin{array}{ccc} x & y & z \end{array}\right]$ refer to origin coordinates of moving platform

(16) $$\begin{array}{c} g_{d}=\left[\begin{array}{cc} R & p\\ 0 & 1 \end{array}\right]=\left[\begin{array}{cc} & x\\ R_{x}\left(\alpha \right)R_{y}\left(\beta \right) & y\\ & z\\ 0 & 1 \end{array}\right], \end{array}$$

where *g*_*d*_ ∈ *SE*(3) is the desired configuration of the tool frame.

The forward kinematics is described in exponential coordinates as

(17) gstθeξ^11θ11eξ^12θ12eξ^13θ13eξ^14θ14eξ^15θ15gst0=eξ^21θ21eξ^22θ22eξ^23θ23eξ^24θ24eξ^25θ25gst0.

Given a desired configuration *g*_*d*_,

(18) $$\begin{array}{c} g_{st}\left(\;\theta \;\right)=g_{d}. \end{array}$$

For branch I, postmultiplying this equation by *g*_*st*_^−1^(0) isolates the exponential maps:

(19) $$\begin{array}{c} e^{{\hat{\xi }}_{11}\theta_{11}}e^{{\hat{\xi }}_{12}\theta_{12}}e^{{\hat{\xi }}_{13}\theta_{13}}e^{{\hat{\xi }}_{14}\theta_{14}}e^{{\hat{\xi }}_{15}\theta_{15}}=g_{d}g_{st}^{-1}\left(\;0\;\right)=g_{1}. \end{array}$$

Apply both sides of (19) to a point *p*_3_ ∈ *R* ^3^ which is the common point of intersection for the universal joint axes (**ξ**_14_, **ξ**_15_). Since $e^{\hat{\xi }\theta }p=p$ if *p* is on the axis of **ξ**, this yields

(20) eξ^11θ11eξ^12θ12eξ^13θ13p13=g1p13,

(21) eξ^13θ13p13=e−ξ^11θ11e−ξ^12θ12g1p13.

Projecting both sides of (21) to the *x*-axis,

(22) 1000eξ^13θ13p13=1000e−ξ^11θ11e−ξ^12θ12g1p13.

*θ*_11_ and *θ*_12_ are eliminated, and we can determine *θ*_13_ as follows:

(23) $$\begin{array}{c} \sin\theta_{13}=\frac{x_{0}-\left(l\cos\alpha \right)/2+\sin\alpha l_{3}+l/2}{-l_{2}}. \end{array}$$

Since *θ*_13_ is known, (20) becomes

(24) $$\begin{array}{c} e^{{\hat{\xi }}_{11}\theta_{11}}e^{{\hat{\xi }}_{12}\theta_{12}}\left(\;e^{{\hat{\xi }}_{13}\theta_{13}}p_{13}\;\right)=g_{1}p_{13}. \end{array}$$

Applying Paden-Kahan subproblem-rotation about two nonintersecting axes [23], we solve for *θ*_11_, *θ*_12_.

The remaining kinematics can be written as

(25) $$\begin{array}{c} e^{{\hat{\xi }}_{14}\theta_{14}}e^{{\hat{\xi }}_{15}\theta_{15}}={e^{-{\hat{\xi }}_{11}\theta_{11}}e^{-{\hat{\xi }}_{12}\theta_{12}}e^{-{\hat{\xi }}_{13}\theta_{13}}g}_{1}. \end{array}$$

Apply both sides of (25) to any point *p* which is not at the intersection of the universal joint axes (**ξ**_14_, **ξ**_15_) as follows:

(26) $$\begin{array}{c} e^{{\hat{\xi }}_{14}\theta_{14}}e^{{\hat{\xi }}_{15}\theta_{15}}p={e^{-{\hat{\xi }}_{11}\theta_{11}}e^{-{\hat{\xi }}_{12}\theta_{12}}e^{-{\hat{\xi }}_{13}\theta_{13}}g}_{1}p. \end{array}$$

Applying Paden-Kahan subproblem-rotation about two subsequent axes, *θ*_14_ and *θ*_15_ are found. So, all *θ*_11_ through *θ*_15_ are determined on branch I. The inverse kinematics of branch II can be solved similarly.

#### 2.4.3. The Workspace of the Mechanism

While solving inverse kinematics, there could be multiple solutions. We need to determine whether the solutions satisfy the constraint conditions. Workspace is considered as a useful measure of the range of the mechanism given the orientation. There are two types of kinematic constraints affecting the available workspace of the mechanism: joint angle limitations and link interference [26]. The joints of animals cannot rotate 360 degrees; thus, the motion is restricted by physical construction. Since the bones of animals have physical dimensions, interference might happen when the mechanism moves. Since links have geometry shapes and physical dimensions, link interference may appear during moving. To keep things simple, assume that every link is cylindrical with the same diameter. The shortest distance between two adjacent links should be greater than the diameter *D*. Let *D*_*i*_ be the minimum distance between the centerlines of two adjacent links. Since *D*_*i*_ is the minimum distance between two line segments, it may not be equal to the common perpendicular segment of the two links (Δ_*i*_). There are the intersection points of two links with their common normal *n*_*i*_. *D*_*i*_ is equal to Δ_*i*_ only if both intersection points are on the links. If one of the intersection points or both are not on the links (i.e., on the extension line), *D*_*i*_ is either the distance of an endpoint of one link to the other link or the distance of the endpoints of two links. The detailed method is discussed in [26].

Thus, the inverse solutions of kinematics are subject to the following constraints:

(27) θmin≤θ≤θmax,Di≥D.

The workspace is divided into slices of thickness Δ*z* parallel to the *XY* plane. As to each slice, the boundary is determined by polar coordinates search method [26] (from a point within the workspace, the angle *φ* is increased by Δ*φ* and the radius *ρ* is increased until the point is out of the workspace). The volume of the reachable workspace is determined by

(28) $$\begin{array}{c} V=\frac{1}{2}\underset{Slices}{\sum }\;\underset{j}{\sum }\rho_{j}^{2}\Delta \varphi \Delta z. \end{array}$$

### 2.5. Parameter Determination

In this paper, we focus on the digits of chamois, which lives in the alpine zone [27]. Alpine terrain has snowy mountains, rough terrain, and alpine meadow [28]. This is very complex terrains for large animals. To live under high alpine conditions, animals have evolved various adaptions [29]. Thus, the feet of chamois may be a complex whole of adaptability. The method above can also apply to other ruminants with similar structure of feet. The average length of the digits from both the manus and the tarsus is shown in Table 1 [30]. Based on the data, we can get the parameters in the mechanism (Table 2), where the distance between two digits (*l*) is an estimate. Due to the lack of concrete data and analysis, we assume that the shortest distance between two adjacent links should be greater than 14 mm, which is greater than the width of proximal phalanx (13.6 mm). Previous measurements of joint angle of goat's foot indicate that the average joint angle of MTP and MCP during stance phase (during level, uphill, and downhill) is 17.6 to 28.6° which is related to the configuration in Figure 3 and the max joint angular excursion of them is 26.1° [13]. While ruminants walk on flat ground, the joints probably will not reach the maximum angle during swing phase and stance phase. The angular range is larger than the measurements during stance phase in case that ruminants go through rugged terrain or other harsh environments. Though we lack the amplitude of lateral angle range of digit joint, we truly know that the range is small. So, we can assume the angular range from reference configuration (***θ*** = 0) in Table 3.

## 3. Result

**Figure 4:**
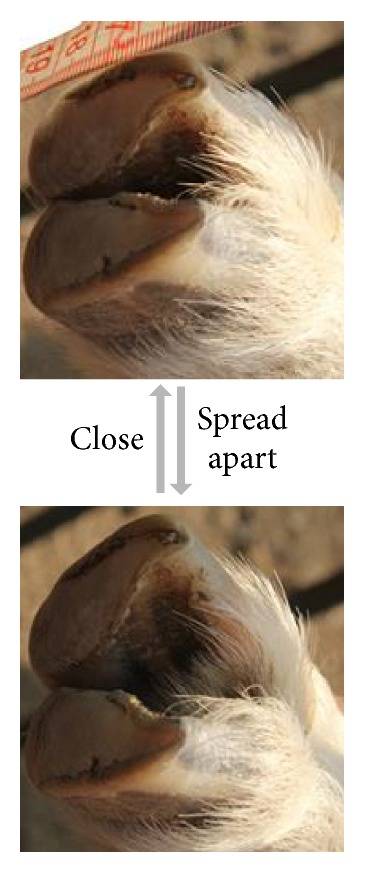
The forefoot of the goat which can close and spread out.

Figure 4 shows that the forefoot of the goat can spread out and close freely. Both forefoot and hindfoot of three goats were examined, demonstrating similar ability.

**Figure 5:**
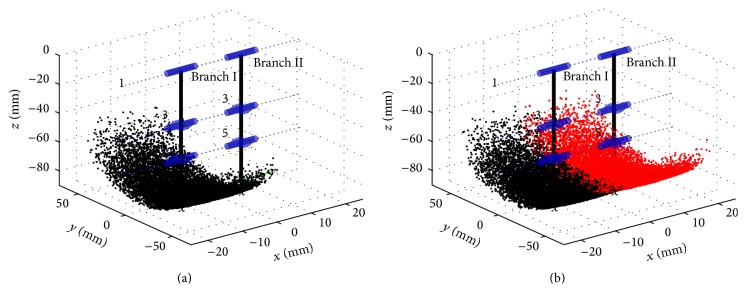
The available workspace of two branches with angle limitation during swing phase. The set of black points in (a) and (b) describes the workspace of branch I, while the red ones in (b) show that of branch II.

We could plug the value of parameters and angle limitation into (3) and (4) using Monte Carlo method. The workspace of two branches can be drawn in Figure 5. Using the method in [31], the workspace volume of branch I is 1.4 × 10^4^ mm^3^.

Figure 5 shows the graphical representation of the workspace of two branches when the two digits are during swing phase. The set of points defines the available workspace that the end-effector of the two branches can reach under joint angle limitations. Each digit could achieve flexion-extension and lateral movements individually. With two endpoints of the digits chosen in the corresponding workspace during swing phase, the attitude of the foot is determined when these two digits step on the ground.

**Figure 6:**
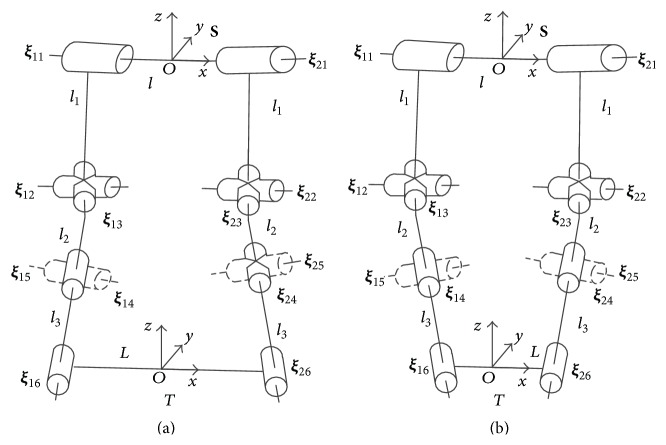
The mechanism of foot while grasping the ground passively. The distance between two endpoints of the digits *L* > *l*, while *θ*_13_ > 0 (relative to the configuration in Figure 3), *θ*_23_ = −*θ*_13_, *θ*_*ij*_  (*i* ≠ 1,3, *j* ≠ 3) = 0 in (a); *L* < *l*, *θ*_13_ < 0 (relative to the configuration in Figure 3), *θ*_23_ = −*θ*_13_, *θ*_*ij*_  (*i* ≠ 1,3, *j* ≠ 3) = 0 in (b).

The single loop mechanism (Figure 3) depicts the foot holding the rock passively during stance phase. The distance between two endpoints of the digits is equal to that between two fetlock joints. Ruminants could also hold other sizes of rock passively, bigger or smaller. Under this definition, one possible configuration is shown in Figure 6. Based on the method given above, the workspace of the mechanism could be determined similarly.

All three mechanisms in Figure 3 and Figure 6 are symmetric around *y*-axis and *z*-axis, so the corresponding workspace has the same shape and size if given the same absolute values of the orientation (*α*, *β*). We choose the values of (*α*, *β*) of the first quadrant.

**Figure 7:**
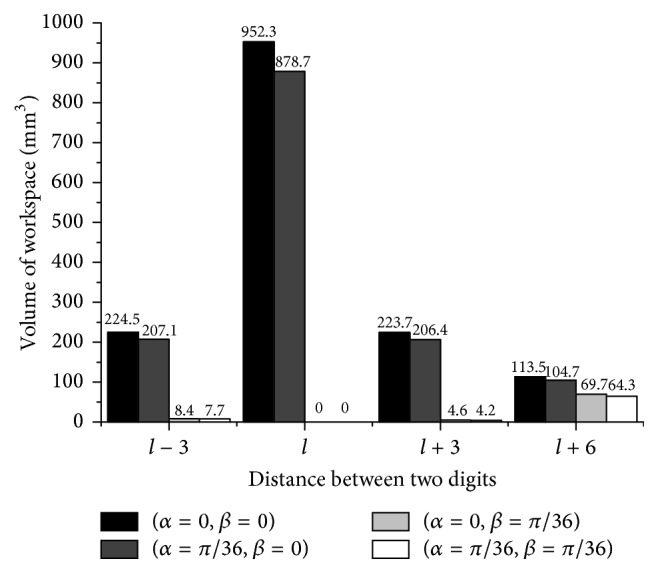
The volume of workspace versus the distance between two digits at four different orientations of the moving platform.

When the foot holds different rock size (let “large rock” represent the condition *L* = *l* + 6, let “big rock” be *L* = *l* + 3, let “normal rock” be *L* = *l*, and let “small rock” be *L* = *l* − 3), the corresponding workspace will change as shown in Figure 7. The workspace volume is much larger at *β* = 0 than at *β* = *π*/36 at different distance between two digits. The increase of the roll angle *α* has little effect on the volume of the workspace (decrease the volume). The workspace of holding “small rock” at *β* = 0 is nearly the same as the one of holding “big rock” at *β* = 0, while the workspace of holding “small rock” at *β* = *π*/36 is larger than the one of holding “big rock” at *β* = *π*/36. The configuration of holding “normal rock” at *β* = 0 shows the largest workspace volume and the configuration of holding “large rock” at *β* = 0 has the smallest workspace volume.

## 4. Discussion

The goal of this study is to investigate possible functions of feet in large ruminants based on similar structure. We give a method that can be used to investigate the functions of all large ruminants. In addition, the mountain habitat of chamois has gradients of aspect, vegetation, altitude, valleys, ridges, edges, and streams [27], which contains various terrains. The foot of chamois somehow has representative among large ruminants. Thus, the result from chamois could give us some general functions of the foot.

Due to the anatomy of the foot (Figure 1), the primary flexion and extension of the foot act like a small limb with parallel hinge joints; the reverse lateral movements (the 3rd and 4th toe bones move at the reverse direction) cause the claws to splay out and close (Figure 4). Figure 5 shows the movement scope of the tip of the foot in swing phase. Given the limitation of the joints, the foot is able to adjust distance between two hooves and the rotation angle (lateral or front-back) before the foot touches the ground. While the volume of the workspace of the horse is only half, the cloven-hoofed animals have more flexible movement to choose the posture of the foot by changing the relative position between two digits. References to earlier papers have described how the cattle or goat goes through the soft terrain [8, 32]; they touch the ground with claws splayed out. Soil or small stones are embedded in interdigital cleft and clamped to provide more contact area, friction angle, and adhesion, produce more propulsion, and reduce subsidence of the foot [7]. When encountering the rock ground, a cloven-claw foot can also grasp the sharp edge of the rock passively. This is similar to how a human grips a stick using two fingers, only passive. Goats and blue sheep tend to splay out their claws when walking downhill to increase the contact area and avoid the slip. The foot is able to adjust distance between two hooves to adapt to different sizes of small stones or rock ledges. Moreover, though the lateral movements of these feet are limited, the foot can tilt laterally by manipulating two digits to reach different heights. This function prevents the ruminants from overturn and improves stability on the cross slope. Only having two digits which can splay out and close can let the ruminants accomplish this adaptability.

After holding a rock or some other bulges passively, two digits in stance phase cannot move as dexterously and freely as the ones in swing phase. The workspace in stance phase is much smaller than the one in swing phase (less than 1000 versus 1.4 × 10^4^ mm^3^). Though the movement of the mechanism is restricted within the workspace in stance phase, the DOF of the mechanism remain 4 or 5. The workspace relative to the base (the top plate of the mechanism) is important when planning a task for the foot. Let *g*_*ts*_ be the configuration of the base frame related to the tool frame (Figure 3) as follows:

(29) $$\begin{array}{c} g_{ts}=g_{st}^{-1}\in SE\left(\;3\;\right). \end{array}$$

Because of the rigid body transformation, the workspace of the top plate relative to the ground is the same as the one which is calculated above. The parallel mechanism could change the position of the carpal joint (Figure 1) when the endpoints of two branches of the parallel mechanism are fixed with the ground. It depicts the ability of the foot to adjust the position of the MCP and MTP (the base frames at the top link in Figure 3) at the given relative orientation when the tip of the digit is fixed with the ground. The volume of the workspace can be used as a measure of the foot dexterity. For ruminants, appropriate foothold can be chosen by the adjustment within the workspace of the foot to regulate the orientation of the limb and the body, even though the tip of the foot is fixed. This function of the foot can help ruminants to adapt to rough terrain and increase stability. Figure 7 shows the influence of the volume of the workspace at different relative orientation when the hooves grasp different size of the object passively and firmly without relative motion. The roll angle *α* represents the movement of flexion and extension of the foot and the pitch angle *β* refers to the lateral movement of the foot. If there are no lateral movements, the workspace is rather large; that is, the foot shows great dexterity under the primary movement of the foot (flexion and extension). Due to the limited lateral movements of the digits, once the lateral movements occurs, the volume of the workspace decreases a lot; that is, the dexterity of foot is weakened at any roll angle *α*. The foot has to sacrifice the dexterity to attain the lateral movements.

Holding different size of rocks passively has an influence on the dexterity of the foot. The excessive distance between two digits (large size rock, *L* = *l* + 6) would incur the loss of the dexterity at *β* = 0. It is because holding large size rock passively needs to open the digits, which already causes lateral movements; that is, the initial joint angle of *θ*_13_, *θ*_23_ is not zero (*θ*_13_ = 3.5°, *θ*_23_ = −3.5°). This occupies part of the angular excursion to retain the configuration. This disadvantage of occupying angular excursion also contributes to the loss of the dexterity when holding small size rock passively (*L* = *l* − 3) or big size rock (*L* = *l* + 3) at the orientation of *β* = 0. In fact, it causes almost the same loss of the workspace volume. However, compared with the reference distance (*L* = *l*), the larger distance (*L* = *l* + 6) provides the largest dexterity at *β* = *π*/36. Actually, with the increased distance, the dexterity augments (*L* > *l*). So, when the lateral movements are needed, holding the larger rock passively would be the better choice to keep both stability and dexterity.

## 5. Conclusion

Therefore, we can summarize several functions of the foot to adapt to the terrain. The foot could change relative position between two digits to splay out or tilt to adapt to the slope in swing phase. The orientation of the foot is prepared for the movement function, that is, grasping a rock passively in stance phase. Holding the rock passively could provide extra foot-ground adhesion force. The simple and similar cloven hoof ensures that the ruminants have some dexterity, even though the tip of the digits is fixed to the ground or the rock. They can choose the proper size of the rock to get greater dexterity at desired orientation in stance phase. These functions facilitate a coherent and stable motion.

These functions are elaborated using kinematics based on screw theory. Many results presented in this paper are exemplified by the data of chamois' foot under certain constrains. We believe that these functions are a basis of terrain adaptation and the general fact which could be found in other ruminants. Based on these common functions, idioadaptive evolution of different ruminants will be detected using similar methods and adding the ligament limitation in future works. Different ruminant species have different length of digits, also the age, the gender, and the different digit will affect the parameters of osseous structures. The difference in length of digits and rotation range of joints may be one of the reasons why ruminants are able to adapt to the different terrains. The terrain adaptability of large ruminants' foot may help to inform the foot design of highly adaptable robot.

## Figures and Tables

**Table 1:** Average length of osseous structures of the digit in chamois (unit: mm).

Species	Proximal phalanx	Middle phalanx	Distal phalanx	Environment

Chamois	38.0	23.3	25.9	Alpine

**Table 2:** Parameter of the mechanism (unit: mm).

Parameters	*l*_1_	*l*_2_	*l*_3_	*l*	*D*

Chamois	38	23.3	25.9	17.525	14

**Table 3:** The angle range of joints (unit: rad).

Joints	*θ*_11_, *θ*_12_, *θ*_15_, *θ*_21_, *θ*_22_, *θ*_25_	*θ*_13_, *θ*_14_, *θ*_23_, *θ*_24_

Angle range	−*π*/6~*π*/6	−*π*/10~*π*/10
